# Complications of Radiofrequency Ablation for Hepatic Hemangioma: A Multicenter Retrospective Analysis on 291 Cases

**DOI:** 10.3389/fonc.2021.706619

**Published:** 2021-07-28

**Authors:** Shilun Wu, Ruize Gao, Tao Yin, Ruhang Zhu, Shigang Guo, Zonghai Xin, Aolei Li, Xinliang Kong, Jun Gao, Wenbing Sun

**Affiliations:** ^1^Department of Hepatobiliary Surgery, Beijing Chaoyang Hospital Affiliated to Capital Medical University, Beijing, China; ^2^Department of General Surgery, Affiliated Hospital of Chifeng University, Chifeng, China; ^3^Department of General Surgery, Chaoyang Central Hospital, Chaoyang, China; ^4^Department of General Surgery, Binzhou Second People’s Hospital, Binzhou, China; ^5^Department of General Surgery, Chaoyang Second Hospital, Chaoyang, China; ^6^Department of Hepatobiliary Surgery, Rizhao Central Hospital, Rizhao, China

**Keywords:** hepatic hemangioma, complication, safety, radiofrequency ablation (RFA), multicenter

## Abstract

**Purpose:**

To report the complications of radiofrequency ablation (RFA) for hepatic hemangioma.

**Patients and Methods:**

Investigators from six centers performed RFA for hepatic hemangioma and used a standardized follow-up protocol. Data were collected from 291 patients, including 253 patients with hepatic hemangioma 5 to 9.9 cm in diameter (group A) and 38 with hepatic hemangioma ≥ 10 cm (group B). Technical success, complete ablation, and complications attributed to the RFA procedure were reported. Analysis of variance was used to determine whether the major complication rate was related to tumor size or clinical experience.

**Results:**

A total of 304 lesions were treated in 291 patients. Technical success was achieved without adverse events in all cases. A total of 301 lesions were completely ablated, including 265 of 265 (100%) lesions in group A, and 36 of 39 (92.31%) in group B. The rate of technology-related complications was similar in groups A and B (5.14% (13/253) and 13.16% (5/38), respectively; *P* = 0.121). Moreover, all technology-related complications occurred during the early learning curve period. The rate of hemolysis-related complications in two groups were 83.40% (211/253) and 100% (38/38) (*P =*0.007) and the systemic inflammatory response syndrome-related complications in two groups were 33.99% (86/253) and 86.84% (33/38) (*P*<0.001). There were no delayed complications in either group.

**Conclusion:**

RFA is minimally invasive, safe, and effective for hepatic hemangiomas 5 to 9.9 cm in diameter. More clinical data are needed to confirm the safety of RFA for hepatic hemangiomas ≥ 10 cm.

## Introduction

Hepatic hemangioma is the most common benign tumor of the liver. Hepatic hemangioma is divided into three categories based on diameter: small (< 5 cm), huge (5–9.9 cm), and giant (≥ 10 cm). Most incidentally identified and asymptomatic hepatic hemangiomas do not need medical interventions. However, hepatic hemangiomas ≥ 5 cm likely will continue to grow and cause symptoms. Moreover, the peripherally located hemangiomas posing the risk of life-threatening spontaneous rupture and hemorrhage. Active treatments for the symptomatic-enlarging hemangiomas need to be considered to relieve the symptoms and prevent the lesions from growth (1–4).

In recent years, radiofrequency ablation (RFA) ablation has been increasingly accepted to treat hepatic hemangioma because of its unique advantages, including minimal invasiveness, definite efficacy, high degree of safety, fast recovery, and wide applicability (1). Although preliminary reports suggest that RFA is safe and effective (5–8), these studies included samples that are too small to allow clinicians to clearly establish the true complication rate, especially for rare but potentially serious complications. For every new intervention, it is essential to evaluate the safety and efficacy to obtain an accurate assessment of the risks and benefits and to determine its relative and absolute contraindications.

To permit an objective assessment of the risks and benefits of RFA, we report the complications encountered by members of a large collaborative group from six centers who have performed RFA in a large number of patients with hepatic hemangiomas (291 patients in total).

## Materials and Methods

### Patient Cohort

We retrospectively reviewed the data of consecutive patients with hepatic hemangioma treated by RFA from June 2009 to July 2019. Data were collected from the clinical databases of six hospitals in China: Beijing Chaoyang Hospital affiliated with Capital Medical University, Beijing, China; Rizhao Central Hospital, Shandong, China; Binzhou Second People’s Hospital, Shandong, China; Chaoyang Central Hospital, Liaoning, China; Affiliated Hospital of Chifeng University, Inner Mongolia Autonomous Region, China; Chaoyang Second Hospital, Liaoning, China. This study was approved by the ethics committee of each participating hospital and complied with the Declaration of Helsinki. All patients provided informed consent for review and analysis of their preoperative medical records.

The diagnosis of the hepatic hemangioma was based on two coincidental radiologic findings on contrast-enhanced ultrasound (US), contrast-enhanced computed tomography (CT), or magnetic resonance imaging (MRI). On US images, hepatic hemangiomas present as a homogeneous, round, or oval lesion with well-defined hyperechogenicity, and the likelihood of posterior acoustic enhancement. Other imaging techniques, such as contrast-enhanced CT or MRI, are recommended for confirmation in case of inconclusive ultrasonographic results, or if a giant hemangioma requires treatment. The typical hemangioma appears on CT or MRI scans as a hypointense, well-defined lesion, which after contrast injection shows peripheral nodular enhancement with progressive homogeneous centripetal filling (1).

Inclusion criteria: maximum diameter of the hemangioma > 5 cm; regular follow-up imaging showing tumor enlargement of more than 1 cm on regular follow-up imaging studies within at least 2 years’ observation; persistent hemangioma-related abdominal pain or discomfort with the definite exclusion of other gastrointestinal diseases *via* gastroscopic examination; patients who declined surgical treatment but consented to RFA.

Exclusion criteria: severe coagulopathy (international normalized ratio > 1.5); infection, especially biliary system inflammation; severe failure of a primary organ such as the liver, kidney, heart, lung, and/or brain; concomitant malignant tumors.

Traditionally, hepatic hemangioma is divided into huge (5–9.9 cm) and giant (≥ 10 cm) based on diameter. Moreover, with larger hepatic hemangiomas, the risk of complications is greater. In our study, we classified the hepatic hemangiomas into two groups (5–9.9 cm as group A and ≥ 10 cm as group B) according to tumor size and severity of complications.

### RFA System

Before 2011, the RITA StarBurst Xli-enhanced RF electrode with RF generator (Radiofrequency Interstitial Thermal Ablation Medical System) was used. The RITA system can achieve maximal ablation zones of 7 cm with a single placement of electrodes, with a maximum power of 250 W. After 2011, the internally cooled cluster electrodes Cool-tip ACTC2025 or ACTC1525 electrodes (COVIDIEN, USA) and RF generator (Covidien Healthcare, Ireland) were used for the RFA procedure. With a 2.5 cm exposed tip, the Cool-tip electrodes can produce ablation zones of 4.5 cm with a single placement of electrodes and a maximum power of 200 W. The power and time of ablation were set based on the tumor size and location according to the manufacturer’s instructions.

### RFA Procedure

All patients were fitted with a tracheal tube or laryngeal mask airway while under intravenous anesthesia to control their respiration. Grounding was achieved by attaching two pads to patient’s thighs. Hepatic hemangiomas deeply located in the liver parenchyma were treated by the percutaneous CT-guided approach, whereas subcapsular hepatic hemangiomas were treated by the laparoscopic approach under intraoperative ultrasound guidance. The procedures and strategies of ablation used has been described previously (1, 4). RFA for hepatic hemangiomas does not require an ablative margin of the normal hepatic parenchyma surrounding the tumor. Therefore, the target scope of ablation for hepatic hemangioma is definite and clear, unlike that for malignant neoplasms. Visualization of hepatic vein is easy for the CT-guided approach. And intraoperative US was used routinely in conjunction with the laparoscopic approach to increase the ability to guide the RF electrode placement and avoid vascular injury.

### Perioperative Data

Preoperatively, we collected the following biographical data of the patients: age, sex, medical history, liver function, and the location, size, and number of hepatic hemangiomas on imaging. Intraoperatively, we recorded the operation path, ablation time, total operation time, vital signs, and urine output and color. Postoperatively, we recorded the length of hospital stay, complications, and laboratory examination results.

### Post-Treatment Evaluation

The primary endpoint was safety (complications related to RFA), technical success, which was defined as correct placement of the ablation device into tumors with completion of the planned ablation protocol, and confirmed complete ablation. Secondary endpoints were improvement of symptoms, change in the size of the ablation zone, recurrence of the residual tumor. Complication of treatment was described using the Clavien-Dindo Classification (9).

All patients underwent follow-up contrast-enhanced CT or MRI 1 month after RFA. Complete ablation was defined as the absence of nodular or irregular enhancement adjacent to the ablation zone. Incomplete ablation was defined as the presence of irregular, peripherally enhanced foci in the ablation zone. In cases of complete ablation, CT or MRI examinations were repeated at 6-month intervals as part of the follow-up protocol. In cases of incomplete ablation, repeated RFA procedures were not performed unless progression of the residual tumor was seen on follow-up imaging performed at 6-month intervals.

### Data Analysis

Continuous data were expressed as mean ± SD and compared between groups using the Student’s t-test and analysis of variance. Differences in categorical data were analyzed by the χ^2^ test or Fisher’s exact test. Two-tailed *P* values < 0.05 were deemed significant. Statistical analysis was performed using SPSS version 26.0. Receiver operating characteristic (ROC) curve was conducted by R 3.5.3 software.

## Results

All six centers responded. The number of patients treated in each center ranged from 12 to 167, and a total of 304 lesions were treated in 291 patients (**Table 1**). Patients were divided into groups based on the diameter of the hepatic hemangioma. Group A contained 253 (86.94%) patients with 265 hepatic hemangiomas 5 to 9.9 cm in diameter, whereas group B contained 38 (13.06%) patients with 39 hepatic hemangiomas ≥ 10 cm in diameter. Of the 291 patients, 278 (95.53%) patients had a single lesion and 13 (4.47%) had two lesions. The patients’ demographic characteristics are provided in **Table 2**.

**Table 1:** Summary of patients and lesions treated with RFA according to location and center.

Location and Center	No. of patients	No. of lesions	5–9.9 cm		≥ 10 cm	
			CT - guide	Laparoscopy	CT - guide	Laparoscopy
Chaoyang Hospital, Beijing BeijingBeijing	167	176	54	93	3	26
Peoples Hospital, Binzhou	40	41	21	13	3	4
Central Hospital, Rizhao	16	16	4	12	0	0
Central Hospital, Chaoyang	23	23	6	16	0	1
Second Hospital, Chaoyang	12	12	8	4	0	0
Affiliated Hospital, Chifeng	33	36	6	28	1	1
Total	291	304	99	166	7	32

RFA, radiofrequency ablation.

**Table 2:** The demographic characteristics of 291 patients in the study.

Variables	Size of tumor		*P*-Value
	5–9.9 cm (n = 253)	≥ 10 cm (n = 38)	
Age (y)	49.20 ± 11.16	48.75 ± 10.45	0.943
Gender			0.570
Male	85 (33.60%)	11 (28.95%)	
Female	168 (66.40%)	27 (71.05%)	
Comorbidities, *N* (%)			
Gallbladder polyps	12 (4.74%)	3 (7.89%)	0.670
Hypertension	44 (17.39%)	9 (23.68%)	0.349
Type 2 diabetes mellitus	10 (3.95%)	2 (5.26%)	1.000
Hepatitis B/C	10 (3.95%)	3 (7.89%)	0.499
Hepatic cysts	11 (4.35%)	3 (7.89%)	0.577
Child-Pugh grading, *N* (%)			1.000
Glass A	246 (97.23%)	37 (97.37%)	
Glass B	7 (2.77%)	1 (2.63%)	
No. of tumor			0.868
Single lesion	241 (95.26%)	37 (97.37%)	
Two lesion	12 (4.74%)	1 (2.63%)	
Distribution of lesion, *N* (%)			0.074
Right lobe	134 (52.96%)	25 (65.79%)	
Left lobe	78 (30.83%)	12 (31.58%)	
Both	41 (16.21%)	1 (2.63%)	
Reason for RFA, *N* (%)			0.003
Abdominal pain	73 (28.85%)	21 (55.26%)	
Enlargement of hemangioma	101 (39.92%)	7 (18.42%)	
Abdominal pain and enlargement hemangioma	79 (31.23%)	10 (26.32%)	
Laboratory findings			
Hb (g/L)	135.13 ± 15.87	130.55 ± 14.81	0.097
ALT (U/L)	21.42 ± 20.91	17.72 ± 8.10	0.289
AST (U/L)	23.78 ± 40.15	18.23 ± 4.14	0.659
TBil (μmol/L)	12.53 ± 5.20	13.90 ± 6.91	0.247
BUN (mmol/L)	4.95 ± 1.47	4.84 ± 1.23	0.614
Creatinine (μmol/L)	70.57 ± 36.30	65.33 ± 12.51	0.484
PT (s)	12.18 ± 1.33	12.87 ± 4.68	0.376
APTT (s)	31.38 ± 4.32	32.85 ± 6.65	0.074

Continuous variables are expressed by median ± standard deviation.

Hb, hemoglobin; TBil, total bilirubin; ALT, alanine aminotransferase; AST, aspartate transaminase; BUN, blood urea nitrogen.

PT, prothrombin time; APTT, activated partial thromboplastin time.

For the patients with multiple hepatic hemangiomas, the hepatic hemangiomas not managed were not included statistical range.

Outcome data for the RFA treatment are given in **Table 3**. A total of 198 hemangiomas underwent laparoscopic RFA, whereas 106 hemangiomas located in the deeper liver parenchyma underwent CT-guided percutaneous RFA. RFA was performed successfully in all patients. No technical failure occurred.

**Table 3:** RFA for 304 hepatic hemangiomas.

Variables	Size of tumor		*P*-Value
	5 - 9.9 cm (n = 265)	≥ 10 cm (n = 39)	
Mean diameter before RFA, (cm)	6.72 ± 1.48	12.56 ± 2.28	<0.001
Approach of RFA			0.019
CT-guided approach	99 (37.36%)	7 (17.95%)	
Laparoscopic approach	166 (62.64%)	32 (82.05%)	
Ablation time, (mins)	43.07 ± 26.79	85.82 ± 34.65	<0.001
RFA session			<0.001
Single session	265 (100.00%)	32 (82.05%)	
Two session	0	7 (17.95%)	
Postoperative length of stay (d)	5.73 ± 3.86	9.04 ± 6.44	<0.001
Mean diameter in 1 month follow-up, (cm)	4.05 ± 1.36	8.71 ± 2.13	<0.001

Continuous variables are expressed by median ± standard deviation.

RFA, radiofrequency ablation.

Of the 304 lesions, 265 of 265 (100%) lesions in group A received only a single RFA session, whereas seven of 38 (18.42%) lesions in group B (single lesions 13–20 cm in diameter) received two RFA sessions to minimize the risk of complications attributable to the RFA procedure. Group A had a significantly shorter ablation time than group B (43.07 ± 26.79 min *vs*. 85.82 ± 34.64 min, *P* < 0.001) (**Table 3**).

### Effectiveness of RFA

Of the 304 hepatic hemangiomas, 301 were ablated completely, including 265 of 265 (100%) lesions in group A and 36 of 39 (92.31%) lesions in group B. Three hepatic hemangiomas were incompletely ablated; the diameters of these lesions were 10.4 cm, 12.5 cm, and 12.6 cm, respectively.

### Complications

The perioperative complications and delayed complications during follow-up are summarized in **Table 4**. In accordance with the cause of complications, we classified the perioperative complications into technology-related complications, hemolysis-related complications, systemic inflammatory response (SIR) syndrome-related complications, and others complications.

**Table 4:** Complications after RFA for hepatic hemangiomas of 291 patients.

Complications	Glavien Grade	Size of tumor		*P*-Value
		5 - 9.9 cm (n = 253)	≥10 cm (n = 38)	
**Perioperative complications**				
**Technology-related, *N* (%) (Time)**		13 (5.14%) (2009 - 2015)	5 (13.16%) (2010 - 2012)	0.121
1. Bleeding at the electrode entry site	III	3 (1.19%) (2009 - 2012)	1 (2.63%) (2011)	0.430
2. Rupture of hepatic hemangioma	III	2 (0.79%) (2011 - 2012)	1 (2.63%) (2010)	0.344
3. Damage of adjacent organs				
Lung injury	I	4 (1.58%) (2011 - 2015)	1 (2.63%) (2012)	0.506
Diaphragmatic injury	II - III	3 (1.19%) (2010 - 2015)	1 (2.63%) (2011)	0.430
Esophageal injury	II	0	1 (2.63%) (2010)	0.272
Symptomatic pleural effusion	III	1 (0.40%) (2011)	0	1.000
**Hemolysis-related, *N* (%) (Time)**		211 (83.40%) (2009 - 2019)	38 (100%) (2009 - 2019)	0.007
1. Hemoglobinuria	I	211 (83.40%) (2009 - 2019)	38 (100%) (2009 - 2019)	0.007
2. Anemia^a^	I	26 (10.28%) (2009 - 2019)	14 (36.84%) (2009 - 2019)	<0.001
3. AKI	II - III	0	3 (7.89%) (2011 - 2018)	0.002
**SIR syndrome-related, N (%) (Time)**		86 (33.99%) (2009 - 2019)	33 (86.84%) (2009 - 2019)	<0.001
1. SIR syndrome	I	86 (33.99%) (2009 - 2019)	33 (86.84%) (2009 - 2019)	<0.001
2. Organ dysfunction				
Myocardial dysfunction	II	0	1 (2.63%) (2019)	0.272
ARDS	IV	0	1 (2.63%) (2012)	0.272
**Others, *N* (%) (Time)**				
1. Postprocedural pain	I	15 (5.93%) (2009 - 2019)	7 (18.42%) (2011 - 2019)	0.017
2. Transient hepatic injury^b^	I	37 (14.62%) (2009 - 2019)	12 (31.58%) (2010 - 2019)	0.009
3. Asymptomatic pleural effusion	I	12 (4.74%) (2009 - 2019)	6 (15.79%) (2010 - 2018)	0.023
4. Skin burn	I	1 (0.40%) (2009)	2 (5.26%) (2009 - 2010)	0.046
**Delayed complications, *N* (%) (Time)**				
1. Liver abscess		0	0	–
2. Biliary damages		0	0	–
3. Tumor progression		0	0	–

AKI, acute kidney injury; SIR, systemic inflammatory response; ARDS, acute respiratory distress syndrome.

^a^Hemoglobin < 100 g/L and hemoglobin was normal before surgery.

^b^Glutamate pyruvate transaminase > 80 U/L.

### Technology-Related Complications

The rates of technology-related complications in groups A and B were 5.14% (13/253) and 13.16% (5/38), respectively (*P* = 0.121). All technology-related complications occurred during the early learning curve period of every clinical research center.

Bleeding at the puncture site (Grade III) occurred in four of 291 (1.38%) patients during laparoscopic RFA. The strategy of simultaneously pressing on the bleeding point and managing the bleeding site was adopted, resulting in no conversion to laparotomy.

Tumor rupture occurred (Grade III) in three of 291 (1.03%) patients; these patients had undergone laparoscopic RFA for hepatic hemangiomas located on the surface of the liver. Hemostasis was achieved by blocking the hepatic hilum combined with RFA under laparoscopy. The blood loss in two of the patients with tumor rupture was 600 ml and 900 ml, respectively. Another patient with a 12 cm hepatic hemangioma developed tumor rupture and was converted to open RFA because of a rapid blood loss of 1,400 ml under laparoscopy. After intra- and postoperative transfusion and rehydration therapy, all three patients recovered well.

Five of 291 (1.72%) patients experienced lung injury (Grade I), and four of 291 (1.38%) patients experienced diaphragmatic injury (Grade II-III). All nine of these patients had a hepatic hemangioma near the dome of the right liver lobe and had undergone CT-guided percutaneous RFA. One patient with a 7.5 cm hemangioma underwent thoracoscopic surgery to insert two chest tubes into the pleural space, which were removed 1 week later. The other eight patients were cured by conservative treatment.

One of 291 (0.34%) patients with an 11.0 cm hemangioma in the left lateral liver lobe developed a lower esophageal fistula (Grade II) caused by direct puncture from one of the radiated arrays and the subsequent ablation, but recovered with conservative treatment.

One patient with a 9.8 cm hepatic hemangioma diagnosed with moderate pleural effusion (Grade III) developed obvious chest tightness and suffocation after RFA, and chest radiography revealed right pulmonary pleural effusion associated with lung compression. The symptoms disappeared after pleural puncture drainage and diuretic therapy.

### Hemolysis-Related Complications

The rates of hemolysis-related complications in groups A and B were 83.40% (211/253) and 100% (38 of 38) (*P =* 0.007), respectively. The typical manifestation of hemolysis was hemoglobinuria. In the present study, hemoglobinuria was diagnosed by the results of Hemoglobin (Hb) positive and red blood cells (RBCs) negative, using urine routine analysis (10). Mild hemolysis subsided within 72 h when managed with adequate hydration, whereas severe hemolysis induced other complications, such as anemia and acute kidney injury (AKI).

In group B, three patients with hemoglobinuria developed AKI (Grade II-III). Two patients (with a 13.6- and 13.7-cm hepatic hemangioma, respectively) presented with obvious hemoglobinuria and progressive elevation of creatinine after RFA. After 1 week of symptomatic treatment comprising the administration of adequate fluids, urine alkalizer, and diuretics, the hemoglobinuria disappeared and the renal function and urine volume of these two patients returned to normal. One patient experienced hemoglobinuria after RFA of a 14.8 cm hepatic hemangioma and subsequently developed oliguria and anhelation, indicating AKI. After 15 days of hemodialysis, the patient’s kidney function returned to normal and he was discharged 27 days after the operation.

Of the patients with hemoglobinuria (Grade I), anemia (Grade I) occurred in 26 of 198 (13.13%) in group A and 14 of 38 (36.84%) in group B. In group A, all 26 patients had slight anemia with no obvious clinical symptoms and did not need treatment. In group B, four of 14 patients had moderate anemia, which was successfully treated with rehydration.

### SIR Syndrome-Related Complications

SIR syndrome has been described in previous articles (9, 11). SIR syndrome (Grade I) was identified in 83 (33.99%) patients in group A and 33 (86.64%) patients in group B (*P <* 0.01). In group B, one patient with SIR syndrome developed myocardial dysfunction (Grade II) and another developed acute respiratory distress syndrome (ARDS) (Grade IV) immediately after RFA. Moreover, we further constructed the ROC curve (**Figure S1**) and detected the cutoff value for tumor size in predicting the presence of SIR syndrome. Optimal cutoff value for tumor size is 7.450 cm (*P* < 0.001, specificity = 0.892, sensitivity = 0.817, area under the ROC curve is 0.887).

### Other Complications

The other complications are reported in **Table 4**. Twenty-two of 291 (7.56%) patients developed postoperative pain (Grade I) that lasted more than 3 days; the pain was mild, and was relieved after the application of common analgesic drugs and antibiotics, without causing serious physical or psychological discomfort. Eighteen of 291 (6.19%) patients developed pleural effusion (Grade I); all patients were asymptomatic, and the pleural effusion was absorbed within 1 week after conservative treatment. Three patients in group B had skin burns (Grade I) at the edge of the grounding pads; these burns healed spontaneously. Transient hepatic injury (Grade I) after ablation was documented in 37 of 253 (14.62%) patients in group A and 12 of 38 (31.58%) in group B; the liver function recovered spontaneously within 1 week.

### Follow-Up

The mean follow-up period was 35 ± 29 months (range 6–120 months). There was no mortality related to RFA, no recurrence, and no delayed complications, such as local tumor progression, biliary damage, or liver abscess formation. No patient developed new symptoms attributed to hepatic hemangioma. The subjective health status and quality of life were rated as good to excellent by 100% of the patients at final follow-up.

## Discussion

In recent years, minimally invasive, local ablation therapies have been increasingly used as an effective alternative treatment for hepatic hemangioma, among which RFA is the most widely utilized treatment modality. Regardless of the resulting benefits, new treatments are always accompanied by unpredictable risks. It is important to characterize these risks and determine methods with which to avoid complications. Hence, multicenter experience of several years is required to characterize special and rare complications and to objectively quantify the expected complication rate. Although the complications caused by needle placement in RFA for hepatic hemangioma are expected to be similar to those of RFA for hepatocellular carcinoma, the complications specific to thermal ablation of hepatic hemangioma still require evaluation in a large population. Significantly, in this study, complications of hepatic hemangioma treated with RFA were recorded and evaluated from the initial development of technology to maturity, which is expected to provide reference for the other research teams to carry out and optimize the RFA techniques.

RFA is a complicated procedure, and substantial experience is required for it to be performed safely. However, in this study, some of the participating centers with less practical experience reported no serious complications. Many of the serious complications mainly occurred at the largest participating centers, which may be attributed to the fact that the center with the most experience was the first to perform RFA and so reported a greater number of complications during a learning curve period in which many of the relative contraindications were identified. Furthermore, with the exception of the first center to perform RFA for hepatic hemangioma, all investigators were required to observe RFA performed in a minimum of 10 patients at a center with more experience prior to commencing RFA at their own institution, which may provide the critical threshold knowledge to master the RFA technique. In addition, many of the smaller centers performed RFA only in straightforward cases, and referred more difficult cases to the larger centers.

A few rare complications were observed in the present study. Hence, we think that the large number of patients analyzed in the present study is almost certainly sufficient to determine the relative risks of RFA for hepatic hemangioma. In accordance with the cause of complications, we classified the main complications into three categories: technology-related, hemolysis-related, and SIR syndrome-related. Technology-related complications mainly comprised bleeding at the electrode entry site, rupture of the hepatic hemangioma, and damage of adjacent organs, such as the esophagus, diaphragm, and lung. All technology-related complications occurred in the early learning curve period. In the later period, technology-related complications can be largely avoided by upgrading ablation equipment, improving techniques, and optimizing ablation strategies (1, 4, 11, 12). For instance, we employed internally cooled cluster electrodes instead of multitined expandable electrodes after 2011. The internally cooled cluster electrode was proved to be more suitable for RFA of hemangioma because of its efficiency and more concentrated release of heat. Besides, internally cooled cluster electrodes keep a steady high temperature in the tumor while limiting vascular cooling. This characteristic increases the effectiveness of perivascular ablation. Moreover, the design of internally cooled cluster electrodes permits their ready placement into target lesions without risk of accidental injury to adjacent organs (13).

The nearly unavoidable hemolysis after RFA, attributable to the generous blood supply of hepatic hemangiomas, especially for those ≥ 10 cm is a major disadvantage of RFA. The hemolysis-related complications, mainly including hemoglobinuria, anemia, and mild renal failure, were the direct results of hemolysis and their severity was directly proportional to the extent of hemolysis. Hb is released upon erythrocyte destruction and is filtered by the glomerulus into the urinary space. In the urinary space, hemoglobin is degraded and releases heme pigments, which can cause tubular injury. Furthermore, volume depletion enhances both vasoconstriction and the formation of obstructing casts, and is of critical importance for the development of hemolysis-induced AKI (14). In the present study, we used strict diagnostic criteria to accurately evaluate hemoglobinuria (10). Therefore, the incidence of hemoglobinuria in this study was relatively high. However, in most cases, hemoglobinuria was minor and disappeared within 3 days. For the hepatic hemangioma 5–9.9 cm in diameter, all the hemolysis-related complications are minor (Grade I).

SIR syndrome-related complications mainly included cases of SIR syndrome and subsequent myocardial dysfunction and organ dysfunction. In the early study period, we thought that postoperative fever was related to the amount of tissue necrosis. However, with the occurrence of some serious complications, such as ARDS and myocardial dysfunction, we found that SIR syndrome played an important role in the occurrence of complications. SIR syndrome is the body’s excessive defensive stress response to inflammatory cytokines, which eventually transforms into a clinical syndrome of pathological systemic inflammation. Mild cases of SIR syndrome are mainly manifested as fever, tachycardia, and tachypnea, whereas severe cases often lead to multiple organ dysfunctions (8). According to experimental study (15), damage to RBCs caused by RFA not only leads to hemoglobinuria but also releases heme to the peripheral circulation, which induced the production of inflammatory factors that contribute to SIR syndrome. So, recognition of hemolytic processes during this treatment will likely serve as a foundation for developing new approaches, to diminish or neutralize the effects of the extracellular Hb and heme. For hemangiomas 5-9.9 cm, using a more effective RFA system can help reduce the RBC damage. In addition, the power and time of ablation are supposed to be based on the tumor size and location according to the manufacturer’s instructions. Patients with hepatic hemangiomas ≥ 10 cm should be sufficiently hydrated to decrease the Hb concentration in the circulation system before and during RFA procedure. When any signs or symptoms indicating hemolysis emerge in the course of RFA, such as rising body temperature and hemoglobinuria, the procedure should be terminated and a repeat RFA treatment may need to be rescheduled based on a comprehensive evaluation of the tumor.

The other complications of RFA for hepatic hemangioma included mild pain, liver damage, asymptomatic pleural effusion, and skin burn injury at the site where the grounding pad was attached. Most patients experienced postoperative pain, mainly from percutaneous puncture and trocar port insertion. Delayed pain that occurred more than 3 days after the procedure was uncommon, and suggested the possibility of more serious underlying complications. Skin burns were noted particularly early in the study period when insufficient grounding pads were used. To prevent such burn injury, patients receiving prolonged RFA require multiple grounding pads or the application of an ice pad to cool the skin in contact with the grounding pad. For superficial lesions, it is important to ensure that the entire active electrode tip is well embedded in the liver and does not course through the skin, muscle, or diaphragm.

It should be emphasized that the morbidity rate patients with hemangiomas 5 to 9.9 cm could be regarded as being acceptable with consideration of the composition of the complications and the benefit of minimal invasiveness patients gained. In view of the present data with high rate of complication, even though with no mortality, we have recognized that RFA for hepatic hemangiomas ≥ 10 cm, be it percutaneous or laparoscopic, is inappropriate. The present study showed that RFA was safe and effective for hepatic hemangiomas less than 10 cm in diameter. More clinical cases and evidence are needed to prove the safety of RFA for hepatic hemangiomas ≥ 10 cm because of the relatively high incidence and severity of hemolysis-related and SIR syndrome-related complications. Many problems associated with RFA of hepatic hemangiomas ≥ 10 cm need further study, such as the optimization of ablation strategies to reduce the incidence of ablation-related complications, the mechanism of some serious complications, and a comparison of the efficacy of RFA *versus* microwave ablation for hepatic hemangiomas ≥ 10 cm.

## Conclusion

The present study supports the use of RFA as an alternative treatment for symptomatic hepatic hemangioma with a diameter of 5 to 9.9 cm because of the low risk of complications and high likelihood of complete ablation. More clinical data are needed to confirm the safety of RFA for hepatic hemangiomas ≥ 10 cm.

## Data Availability Statement

The original contributions presented in the study are included in the article/**Supplementary Material**. Further inquiries can be directed to the corresponding authors.

## Ethics Statement

This study was approved by the ethics committee of Beijing Chaoyang Hospital affiliated with Capital Medical University, Beijing, China; Rizhao Central Hospital, Shandong, China; Binzhou Second People’s Hospital, Shandong, China; Chaoyang Central Hospital, Liaoning, China; Affiliated Hospital of Chifeng University, Inner Mongolia Autonomous Region, China; Chaoyang Second Hospital, Liaoning, China and complied with the Declaration of Helsinki. All patients provided informed consent for review and analysis of their preoperative medical records. The patients/participants provided their written informed consent to participate in this study.

## Author Contributions 

SW and RG: wrote the manuscript and prepared the tables. TY, RZ, SG, ZX, AL, and XK modified the manuscript. JG and WS designed the manuscript. All authors contributed to the article and approved the submitted version.

## Funding

This study was supported by Beijing Municipal Administration of Hospitals Incubating Program (PX2020011).

## Conflict of Interest

The authors declare that the research was conducted in the absence of any commercial or financial relationships that could be construed as a potential conflict of interest.

## Publisher’s Note

All claims expressed in this article are solely those of the authors and do not necessarily represent those of their affiliated organizations, or those of the publisher, the editors and the reviewers. Any product that may be evaluated in this article, or claim that may be made by its manufacturer, is not guaranteed or endorsed by the publisher.

## Supplementary Material

Supplementary Figure 1 ROC curve for tumor size in predicting the presence of SIR syndrome. Optimal cutoff value for tumor size is 7.450 cm (*P* < 0.001, specificity = 0.892, sensitivity = 0.817, area under the ROC curve is 0.887).
