# Supplementary figures and images for: Complications of Radiofrequency Ablation for Hepatic Hemangioma: A Multicenter Retrospective Analysis on 291 Cases
Source: Front Oncol. 2021 Jul 28;11:706619. doi: 10.3389/fonc.2021.706619 (PMC8356044; doi:10.3389/fonc.2021.706619)

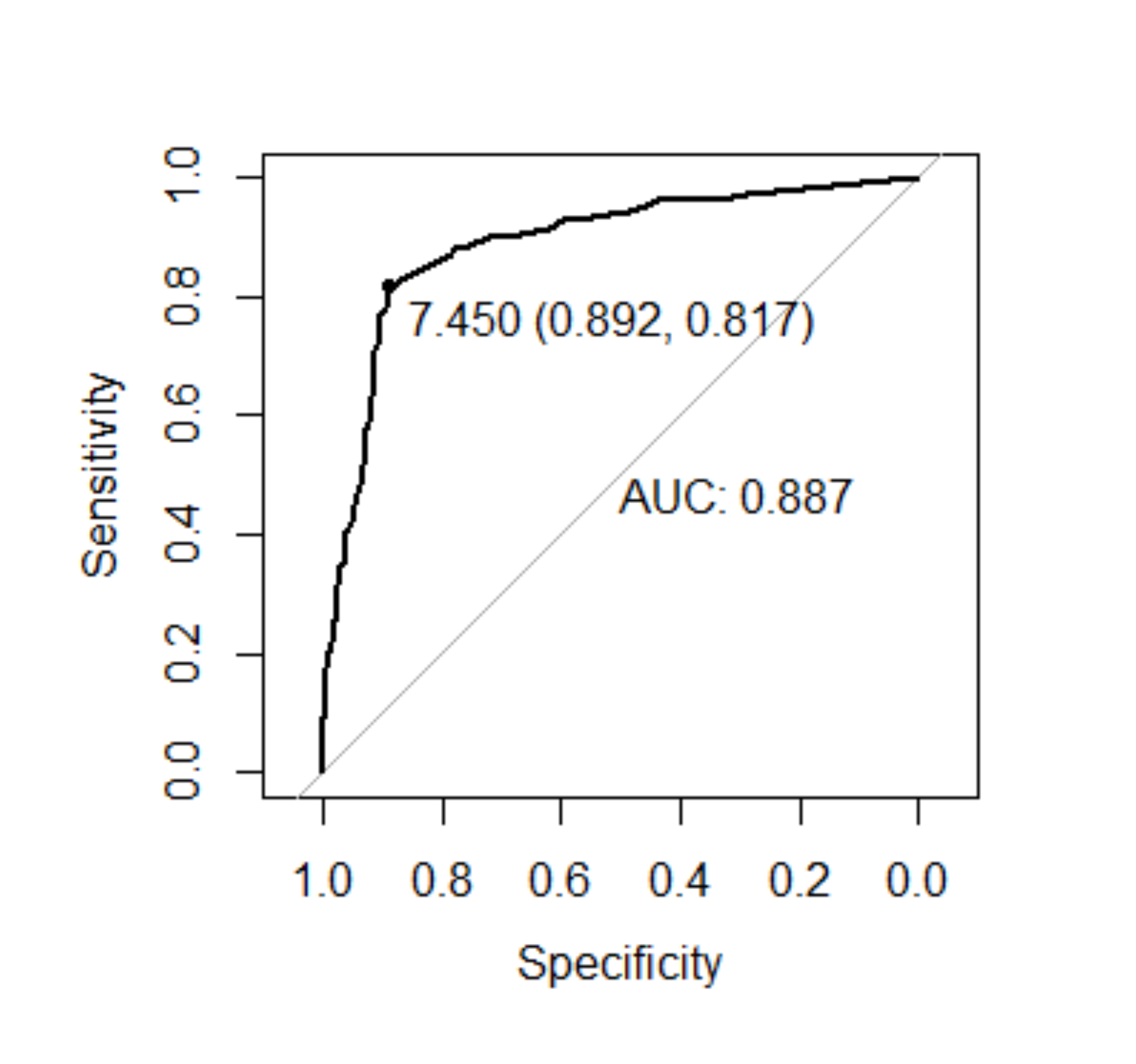

Supplement: Supplementary Figure 1 — ROC curve for tumor size in predicting the presence of SIR syndrome. Optimal cutoff value for tumor size is 7.450 cm (P < 0.001, specificity = 0.892, sensitivity = 0.817, area under the ROC curve is 0.887). [file Image_1.tif]
